# CCRT and aerobic exercise: a randomised controlled study of processing speed, cognitive flexibility, and serum BDNF expression in schizophrenia

**DOI:** 10.1038/s41537-022-00297-x

**Published:** 2022-10-20

**Authors:** Yuanyuan Dai, Hongyan Ding, Xiaozi Lu, Xiumei Wu, Chunhua Xu, Tingting Jiang, Liang Ming, Zhong Xia, Chuanfu Song, Hongxian Shen, Wei Hao, Shucai Huang

**Affiliations:** 1 The Fourth People’s Hospital of Wuhu, Wuhu, 241000 China; 2 Qingdao Mental Health Center, Qingdao, 266034 China; 3 National Clinical Research Center for Mental Disorders, and Department of Psychiatry, The Second Xiangya Hospital of Central South University, Changsha, 410011 China

**Keywords:** Schizophrenia, Psychosis

## Abstract

Computerised cognitive remediation therapy (CCRT) and aerobic exercise are often used to rehabilitate social functioning in patients with schizophrenia. However, there is limited knowledge regarding the effects of CCRT combined with aerobic exercise on cognitive function and brain-derived neurotrophic factor (BDNF) levels in patients with schizophrenia and cognitive impairment. Ninety-six patients with schizophrenia and cognitive impairment were included in this study and randomly divided into control, aerobic exercise (AE), and CCRT combined with aerobic exercise (CAE) groups. Changes in processing speed and cognitive flexibility at week 8 were evaluated as primary and secondary cognitive outcomes using the Trail Making Test: Part A, the Brief Assessment of Cognition in Schizophrenia: Symbol Coding Test, and the Stroop Colour-Word Test. Positive and Negative Syndrome Scale (PANSS) scores and serum BDNF expression were determined as other secondary outcomes. The CAE group showed significantly better performance in terms of changes in processing speed and cognitive flexibility than the control and AE groups at week 8 (*p* < 0.05); however, no significant improvements in processing speed and cognitive flexibility were found between the control and AE groups. The CAE group showed significant improvements in the PANSS negative symptoms than the control group at week 8 (*p* < 0.05), but the AE group showed no significant difference in the changes of PANSS negative symptoms when compared with the other two groups. The CAE group and AE group showed a greater increase in serum BDNF levels than the control group (*p* < 0.01), but there was no significant difference in serum BDNF expression between the CAE group and AE group. In conclusion, 8-week CCRT combined with aerobic exercise may improve some cognitive performance and negative symptoms in patients with schizophrenia. Aerobic exercise may have an immediate effect on serum BDNF levels rather than cognitive function.

## Introduction

Cognitive impairment is a common symptom of schizophrenia. More specifically, cognitive impairment is considered to be the third symptom component of schizophrenia (in addition to negative and positive symptomology). This symptom component mainly involves obstacles within attention, memory, thinking, and information integration processes^1,2^.

Cognitive deficits severely undermine the long-term prognosis of patients with schizophrenia. More specifically, even if hallucinations, delusions, and other psychotic symptoms improve, these cognitive deficits will continue to hinder the patient’s recovery of social function; this will eventually lead to difficulties for the patient to return to society^3,4^. Therefore, treatment of cognitive deficits in patients with schizophrenia is of great clinical significance.

Previous clinical research evidence has shown that the amelioration of cognitive deficits via antipsychotic pharmacological treatments in patients with schizophrenia is currently extremely limited^5^, and that the efficacy of other pharmacological drugs addressing these cognitive deficits also seems to bemarginal^6^.

In clinical practice, aerobic exercise is often used as an adjuvant therapy to improve cognitive, behavioural, and social functioning in patients with schizophrenia. Previous studies have reported that exercise may have a positive impact on working memory, motor speed, attention, and executive function^7–9^. Structured physical exercise is also considered to ameliorate psychotic symptomology^10,11^. For example, some studies have found that exercise may significantly improve the negative symptoms, including depressive symptoms, in patients with schizophrenia, and improve their social function and quality of life^12,13^. In addition, physical exercise may reduce the risk of physical diseases in schizophrenia patients by improving cardiopulmonary and metabolic functions^14^, and previous studies have found that physical exercise may increase hippocampal volume and white matter integrity in patients with schizophrenia^15,16^. It has also been reported that physical exercise is associated with higher levels of neurotrophic factors, larger grey and white matter volumes, and better cognitive abilities in patients with schizophrenia^17,18^.

Currently, computerised cognitive remediation therapy (CCRT) is considered to have a definitive positive effect on the improvement of cognitive function in patients with schizophrenia, including with regard to attention, memory, and executive functioning^19–21^. However, little is known about the underlying biological mechanisms mediating these associations.

Brain-derived neurotrophic factor (BDNF) is widely distributed in the brain, plays an important role in the structure and function of neurons by catalysing the growth and reorganisation of dendritic spines in response to changes in neuronal function, and is of great significance to the maintenance and development of cognitive functions^22,23^. Studies have found that serum BDNF levels in adult schizophrenia patients are usually lower than in normal controls^24–26^. Moreover, some brain function defects may be related to the disturbance of the BDNF system seen in patients with schizophrenia, including a decreased number of BDNF-positive neurons and a lower level of BDNF in the cortex and hippocampus^27,28^. In addition, research to date indicates that serum BDNF levels may be a potential biomarker for evaluating the effects of cognitive training^23^. Limited evidence also shows that exercise training increases the expression of BDNF in the brain and peripheral blood, which may have a positive effect on cognitive functioning^29,30^.

Therefore, this study aimed to explore the effects of CCRT and aerobic exercise on information processing speed, cognitive flexibility, and BDNF in patients with schizophrenia presenting with cognitive impairment.

## Patients and methods

### Study design

This was a longitudinal, randomised, single-blind trial conducted at the Fourth People’s Hospital of Wuhu (Wuhu, China). This study was registered at the Chinese Clinical Trial Registry (ChiCTR2000035746).

### Study hypotheses

Our primary hypothesis was that CCRT and aerobic exercise would improve brain information processing speed and cognitive flexibility in patients with schizophrenia. Our secondary hypothesis was that an improvement in cognitive performance would be accompanied by an increase in serum BDNF levels.

### Sample size

Based on a similar study conducted in schizophrenia patients^31^, we estimated a necessary sample size of 69 (23 per group) according to the superiority test formula (${{n}} = \frac{{\psi ^2(\mathop {\sum}\nolimits_{j = 1}^k {S_j^2/k} )}}{{\mathop {\sum}\nolimits_{j = 1}^k {(X_j - X)2/k - 1} }}$, wherein *k* represents the number of groups, and *X*_*j*_ and *S*_*j*_ represent the mean value and standard deviation of each group, respectively) for multiple groups^32^, with *α* = 0.05, *β* = 0.20, and *k* = 3. Within our enrolment process, the sample size was increased by 30% in consideration of potential study dropouts; hence, at least 90 participants were required (30 per group). Ultimately, 96 participants were recruited through in-hospital advertising in this study.

### Participants and inclusion/exclusion criteria

All participants were inpatients at the Fourth People’s Hospital of Wuhu and met the following inclusion criteria: (1) age 18–55 years; (2) patients meeting the Diagnostic and Statistical Manual of Mental Disorders, Fifth Edition criteria for schizophrenia, with a duration of diagnosis of >2 years; (3) hallucinations, delusions, and other positive psychotic symptoms had disappeared (Positive and Negative Syndrome Scale [PANSS] positive score <14); (4) Montreal Cognitive Assessment (MoCA) score of <26^33^; and (5) more than 5 years of educational experience, with a certain level of language comprehension so as to ensure that the participants could understand and sign the informed consent form and follow the prompts to perform simple mouse operations. The exclusion criteria were as follows: (1) suffering from organic diseases that may affect cognitive function; (2) taking non-antipsychotic drugs that may affect cognition; and (3) definite organic or functional damage to the respiratory, circulatory, and motor systems.

### Randomisation

A standard online randomisation programme was used to randomly assign participants to control, aerobic exercise (AE), and CCRT combined with aerobic exercise (CAE) groups after obtaining informed consent and checking for eligibility (http://www.randomization.com).

### Blinding

The blinding principle was applied as much as possible in this study. Namely, the participants in the AE and CAE groups were mixed for the aerobic exercise interventions, and all guidance coaches (nurses) were unaware of the participants’ groupings, all investigators in charge of cognitive evaluation and serum BDNF concentration detection were blinded to study group assignment, and the investigators supervising data monitoring and statistical analysis were also not informed as to the study group assignments. At the same time, we tried to incorporate objective cognitive test data as evaluation indicators in order to reduce the interference of subjective factors pertaining to both participants and researchers on the study results. The nature of the intervention meant that the participants could not be blinded to their intervention group assignment. However, they were required to keep their group assignment confidential from their raters and therapists.

### Intervention setting

#### Intervention as usual

Clinical intervention continued for all study participants in accordance with the Chinese guidelines for the prevention and treatment of schizophrenia^34^. Usual treatment included antipsychotic pharmacological treatment, psychological consultation, medical care, and behaviour modification.

#### Aerobic exercise training

The complete intervention duration of the aerobic exercise training and CCRT interventions was 8 weeks. Participants in the AE and CAE groups were provided 45-min sessions of aerobic exercise by qualified instructors from Monday to Friday. The aerobic physical exercise protocol was designed based on previous reports by Lin et al.^35^ and Erickson et al.^36^, with minor modifications: music warm-up exercise (10 min) and walking on a treadmill at a speed of 6–9 km/h (30 min) followed by cool-down stretching (5 min). Heart rate was monitored during the exercise sessions using a portable recorder so as to ensure that participants maintained a moderate-intensity exercise exertion level of 50–70% of their maximum heart rate^36^. Blood pressure was recorded before and after training.

#### CCRT training

Eight weeks of CCRT training was provided in the CAE group. The CCRT system administered herein was developed according to the principles of Wykes and Reeder^37^. This system consists of 30 exercise sequences that dynamically adjust the difficulty as the accuracy reaches 80%, and is administered within two 30-min sessions each week for a total of 8 weeks (i.e., 16 total sessions). This intervention was supervised by experienced therapists at a ratio of one therapist to five participants.

### Assessments and outcomes

All clinical interviews were conducted by three licenced psychiatrists with MDs who had at least 5 years of clinical experience in psychiatry. The following clinical data were evaluated at baseline using self-developed questionnaires (week 0): demographic data, medical history, and clinical medications (including age, sex, education, duration of illness, accumulated duration of medication use, use of psychiatric drugs, and smoking); psychotic symptoms were evaluated using the Chinese version of the PANSS^38^; and cognitive impairment screening was conducted using the Chinese version of the MoCA.

Processing speed was assessed using the Trail Making Test: Part A (TMT-A) and the Brief Assessment of Cognition in Schizophrenia: Symbol Coding Test (SCT)^39^, cognitive flexibility was evaluated using the Stroop Colour-Word Test (SCWT; with scores based on the number of completed categories over 45 s)^40^. Serum BDNF concentrations were detected at baseline using standard methodology^41^. Evaluations of processing speed and cognitive flexibility and the detection of BDNF concentrations in serum were repeated at mid-training (week 4), end of training (week 8), and follow-up (week 12).

#### Outcome measures

The primary outcomes evaluated in this study included two cognitive domains. The primary cognitive outcome was the change from baseline in processing speed at week 8, and the secondary cognitive outcome was processing speed at weeks 4 and 12 and cognitive flexibility at weeks 4, 8, and 12.

Other secondary outcomes were the change in serum BDNF level at weeks 4, 8, and 12 and PANSS scores at week 8. The detection process with regard to serum BDNF was as follows. Blood was withdrawn into single-use vacuum tubes between 06:30 am and 08:30 am after an overnight fast. In order to maintain sequential treatment, we did not impose restrictions on the patients regarding their use of medications before blood withdrawal. Serum was extracted from blood samples and stored at –80 °C until the time of assay. All collected serum samples were assayed using an enzyme-linked immunosorbent assay. The BDNF Emax ImmunoAssay system (Promega G7610, Madison, WI, USA) was used by one technician who was blinded to the study grouping. Evaluations took place in one laboratory and were conducted according to the manufacturer’s instructions, as described in a previous study^41^. Briefly, an anti-BDNF monoclonal antibody was used to coat 96 well microplates, which were then incubated overnight at 4 °C. Blocking buffer was added, and the samples were incubated for 1 h at 16 °C. The serum samples were diluted 50 times with assay buffer, following which the diluted samples and BDNF standard were placed in microplates with horizontal shaking for 2 h at 16 °C and were washed with buffer containing Tris-buffered saline and Tween 20. The microplates were incubated with an anti-human BDNF polyclonal antibody for 2 h at 16 °C and were then washed in the buffer. The microplates were then incubated with horseradish peroxidase-conjugated anti-immunoglobulin Y antibody at 16 °C for 1 h, washed with buffer, and incubated with the peroxidase substrate tetramethylbenzidine solution for 10 min to induce a colour reaction. The reaction was terminated using 1% hydrochloric acid. Absorbance was measured at 450 nm with an automated microplate reader.

### Safety monitoring

The trial was approved by the Medical Ethics Committee of the Fourth People’s Hospital of Wuhu, and both the participants and their guardians signed an informed consent form before the study participation. All participants underwent the necessary physical examinations to ensure that they were fit to participate in aerobic training and CCRT, and their physical condition was re-examined every two weeks until the end of the follow-up. All written data materials were stored in a secure cabinet equipped with two locks, and electronic data were stored in a password-protected computer and in a special password-protected database. External experts from the ethics committee monitored the progress of the project every month.

### Data analysis

All data were analysed using SPSS statistical software (version 20.0; IBM Corp., Armonk, NY, USA). The data were manually entered into an SPSS database after independent quality control checks were conducted by two researchers. The data were also scrutinised to identify out-of-range values; any such values were examined and discrepancies were corrected within the source files.

One-way analysis of variance (ANOVA) and *χ*^2^ tests were used to compare continuous and categorical data among the three groups as appropriate. Nonparametric tests were used when the assumptions of normality and/or homogeneity of variance were violated. Repeated measures ANOVA was performed to analyse potential effects on outcomes, with the treatment group (aerobic exercise combined with CCRT, aerobic exercise, or control groups) considered as an intergroup factor and time (baseline, 4 weeks, 8 weeks, and 12 weeks) considered as an intragroup factor. Age, education, gender, disease duration, and baseline values of the outcome measure were treated as covariates. Post hoc multiple comparisons at different evaluation times were performed using the Bonferroni–Dunnett test. Pearson’s correlation analysis was conducted to investigate relationships between the changes in cognitive and non-cognitive outcomes (i.e., serum BDNF level and PANSS score). For the two-tailed test, the level of statistical significance was set to *p* < 0.05.

## Results

Ninety-six participants were eligible to participate in the trial. Of these, 14 individuals withdrew from the trial for personal reasons without undergoing CCRT or AE training (Control 1, CAE 6, AE 7). The reasons for withdrawal included (1) discharge before the trial started (CAE 1, AE 2); (2) worsened symptoms (Control 1, AE 1); (3) unwillingness to rush to the training room within the agreed-upon time (CAE 1; AE 2); (4) participants’ concern about not being able to complete computer operations (CAE 3); and (5) quitting without a specified reason (CAE 1; AE 2). A total of 82 participants were included in the final analysis based on the intention to treat principle.

### Demographic and clinical characteristics

Detailed information on demographic and other clinical characteristics is shown in Table 1. There were no differences in age, sex, education, smoking, duration of schizophrenia, or antipsychotic use among the three groups. PANSS scores, TMT-A time, SCT scores, SCWT performance, and serum BDNF levels were similar among the three groups (Table 1).

**Table 1:** Baseline demographic data, clinical variables and cognitive characteristics of patients in three groups (*n* = 82).

	Control subjects	CAE subjects	AE subjects	*F* or *χ*^2^ value	df	*p* value
	(*n* = 31)	(*n* = 26)	(*n* = 25)			
Demographics						
Age (years)	44.06 (8.40)	41.50 (8.72)	41.40 (7.86)	0.947	2,79	0.392
Years of education	9.16 (2.72)	10.00 (2.74)	9.00 (3.16)	0.917	2,79	0.404
Gender (men/women)	20/11	22/4	20/5	3.473	2	0.176
Clinical variables						
Duration of Illness (months)	236.61 (112.71)	236.27 (147.55)	202.24 (98.20)	0.712	2,79	0.494
Duration of taking antipsychotics (months)	203.87 (116.96)	161.62 (129.38)	176.76 (101.13)	0.981	2,79	0.379
Type of antipsychotics (FGA/SGA)	3/28	2/24	3/22	0.296	2	0.874
Equivalent dose of chlorpromazine	374.19 (97.36)	357.69 (102.66)	328.00 (96.91)	1.524	2,79	0.224
Smoking	8 (25.81%)	5 (19.23%)	6 (24.00%)	0.357	2	0.836
PANSS positive score	11.13 (2.54)	12.23 (2.41)	10.84 (2.64)	2.192	2,79	0.118
PANSS negative score	18.71 (4.16)	19.04 (4.84)	17.60 (3.82)	0.791	2,79	0.457
PANSS general psychopathology score	33.87 (4.65)	34.23 (6.26)	35.96 (5.30)	1.136	2,79	0.326
PANSS total score	63.71 (8.41)	65.46 (9.91)	64.12 (7.05)	0.315	2,79	0.731
Cognitive functions						
MoCA score	19.97 (1.54)	20.08 (1.98)	20.44 (2.02)	0.484	2,79	0.618
Trail Making Test-A (s)	90.42 (28.22)	88.54 (25.32)	88.48 (20.98)	0.055	2,79	0.946
Symbol Coding Test	27.32 (11.65)	28.85 (14.39)	29.44 (11.27)	0.218	2,79	0.804
Stroop Word	65.87 (18.37)	65.38 (12.90)	67.04 (18.12)	0.066	2,79	0.936
Stroop Colour	42.26 (14.26)	41.54 (9.29)	43.76 (14.10)	0.199	2,79	0.820
Stroop Colour-Word	21.97 (10.54)	22.15 (9.40)	23.84 (10.48)	0.271	2,79	0.764
BDNF in serum (ng/ml)	20.88 (2.74)	20.28 (3.31)	21.22 (2.44)	0.717	2,79	0.491

Continuous variables are listed as mean (SD).

*AE* aerobic exercise, *CAE* CCRT combined with aerobic exercise, *FGA* first-generation antipsychotics, *SGA* second-generation antipsychotics, *PANSS* Positive and Negative Syndrome Scale, *BDNF* brain-derived neurotrophic sector.

### Treatment outcomes

#### Primary cognitive outcomes

**Fig. 1:**
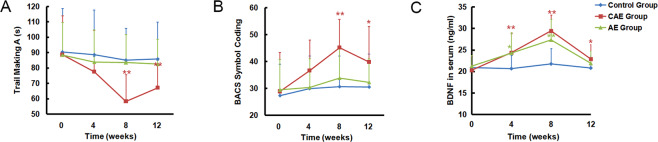
Comparison of processing speed and serum BDNF (*n* = 82). **A** Changes of the Trail Making Test-A; **B** changes of the Symbol Coding Test; and **C** changes of BDNF level in serum. The results are expressed as mean ± SD. Differences between groups were analysed using two-way ANOVA for repeated measures, and post hoc multiple comparisons at different endpoints were performed using Bonferroni–Dunn test. *p ≤ 0.05, **p ≤ 0.01, as compared with control group. BDNF brain-derived neurotrophic factor, AE aerobic exercise, CAE CCRT combined with aerobic exercise.

We found a statistically significant overall difference among the three groups in TMT-A time (*F* = 5.768, *p* = 0.005, η^2^ = 0.127) and SCT scores (*F* = 5.148, *p* = 0.008, η^2^ = 0.115) (Fig. 1A, B). Statistically significant time effects (TMT-A: *F* = 8.976, *p* < 0.001, *η*^2^ = 0.102; SCT: *F* = 15.783, *p* < 0.001, η^2^ = 0.167) and time-by-group effects (TMT-A: *F* = 3.302, *p* = 0.004, η^2^ = 0.077; SCT: *F* = 4.183, *p* = 0.001, η^2^ = 0.096) were also found with respect to processing speed.

The changes in TMT-A (*F* = 7.937, *p* < 0.001, η^2^ = 0.167) and SCT (*F* = 8.882, *p* < 0.001, η^2^ = 0.184) from baseline to week 8 were significantly different among the three groups (Fig. 1A, B and Table 2). Post hoc analysis revealed that the CAE group showed significant improvement in the processing speed test at week 8 as compared with the AE (TMT-A: *p* = 0.04; SCT: *p* = 0.008) or control groups (TMT-A: *p* = 0.002; SCT: *p* < 0.001), and no significant difference was found between the AE and control groups in the change of TMT-A time or SCT scores at week 8.

**Table 2:** Changes of outcomes in three groups after 8 weeks of intervention (*n* = 82).

	Control subjects	CAE subjects	AE subjects	*F* value	df	*p* value	η^2^ value	Group difference
	(*n* = 31)	(*n* = 26)	(*n* = 25)					
Speed of processing								
∆ Trail Making Test-A (s)	–5.26 (27.18)	–30.19 (24.88)	–5.04 (27.19)	7.937	2,79	0.001	0.167	CAE > Control, AE
∆ Symbol Coding Test	3.32 (11.54)	16.27 (9.68)	4.44 (15.77)	8.882	2,79	<0.001	0.184	CAE > Control, AE
Cognitive flexibility								
∆ Stroop Word	0.65 (17.81)	22.61 (14.70)	4.48 (24.88)	9.934	2,79	<0.001	0.201	CAE > Control, AE
∆ Stroop Colour	–0.81 (14.32)	13.85 (8.99)	2.56 (19.14)	7.514	2,79	0.001	0.160	CAE > Control, AE
∆ Stroop Colour-Word	2.16 (4.50)	3.88 (7.11)	2.24 (4.82)	0.823	2,79	0.443	0.020	
PANSS score								
∆ Positive	–0.39 (1.99)	–0.96 (2.29)	0.20 (1.85)	2.044	2,79	0.136	0.049	
∆ Negative	–1.06 (2.37)	–2.69 (1.83)	–1.48 (2.22)	4.202	2,79	0.018	0.096	CAE > Control
∆ General Psychopathology	–1.81(3.64)	–2.23 (3.92)	–1.92 (4.61)	0.081	2,79	0.922	0.002	
∆ Total	–3.23 (4.72)	–5.73 (4.72)	–2.96 (6.20)	2.306	2,79	0.106	0.055	
∆ BDNF in serum (ng/ml)	0.86 (4.77)	9.14 (4.18)	6.09 (5.09)	22.81	2,79	<0.001	0.366	CAE, AE > Control

∆ represents the value difference after 8 weeks of intervention. Continuous variables are listed as mean (SD).

*BDNF* brain-derived neurotrophic factor, *AE* aerobic exercise, *CAE* CCRT combined with aerobic exercise.

#### Secondary cognitive outcomes

The changes in TMT-A (*F* = 3.51, *p* = 0.032, η^2^ = 0.083) and SCT (*F* = 3.881, *p* = 0.025, η^2^ = 0.089) from baseline to week 12 were significantly different among the three groups (Fig. 1A, B). Post hoc analysis revealed that the CAE group showed significant improvement in the processing speed test at week 12 as compared with the AE (TMT-A: *p* = 0.023; SCT: *p* = 0.043) or control groups (TMT-A: *p* = 0.002; SCT: *p* = 0.011), and no significant difference was found between the AE and control groups in the change of TMT-A time or SCT scores from baseline to week 12. However, ANOVA did not reveal any significant difference among the three groups in the change of TMT-A time or SCT scores from baseline to week 4.

**Fig. 2:**
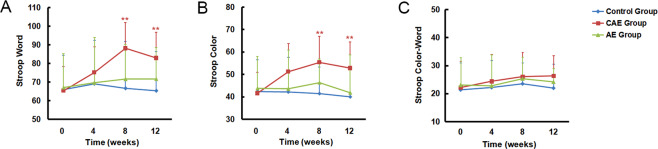
Performance of cognitive flexibility (*n* = 82). **A** Changes of the Stroop Word Test; **B** changes of Stroop Colour Test; and **C** changes of Stroop Colour-Word Test. The results are expressed as mean ± SD. Differences between groups were analysed using two-way ANOVA for repeated measures, and post hoc multiple comparisons at different endpoints were performed using Bonferroni–Dunn test. **p ≤ 0.01, as compared with the control group. AE aerobic exercise, CAE CCRT combined with aerobic exercise.

There was a statistically significant difference across the three groups in Stroop word test scores (*F* = 3.657, *p* = 0.030, η^2^ = 0.085) and Stroop colour test scores (*F* = 5.300, *p* = 0.007, η^2^ = 0.118) (Fig. 2A, B). Significant time effects (Stroop word test: *F* = 8.446, *p* < 0.001, η^2^ = 0.097; Stroop colour test: *F* = 3.894, *p* = 0.010, η^2^ = 0.047) and time-by-group effects (Stroop word test: *F* = 5.178, *p* < 0.001, η^2^ = 0.116; Stroop colour test: *F* = 3.804, *p* = 0.001, η^2^ = 0.088) were also observed.

The changes in the Stroop word test (*F* = 9.934, *p* < 0.001, η^2^ = 0.201) and colour test (*F* = 7.514, *p* = 0.001, η^2^ = 0.160) from baseline to week 8 were significantly different among the three groups (Fig. 2A, B and Table 2). Post hoc analysis revealed that the CAE group showed significant improvement in the Stroop word test (vs AE group, *p* = 0.009; vs control group, *p* < 0.001) and Stroop colour test (vs AE group, *p* = 0.033; vs control group, *p* = 0.001) from baseline to week 8. However, no significant difference was found between the AE and control groups in the change of the Stroop word test or Stroop colour test from baseline to week 8. The changes in the Stroop word test (*F* = 7.445, *p* = 0.001, η^2^ = 0.159) and colour test (*F* = 7.004, *p* = 0.002, η^2^ = 0.151) from baseline to week 12 were significantly different among the three groups (Fig. 2A, B). Post hoc analysis revealed that the CAE group showed significant improvement in the Stroop word test (vs AE group, *p* = 0.038; vs control group, *p* = 0.001) and Stroop colour test (vs AE group, *p* = 0.007; vs control group, *p* = 0.003) from baseline to week 12. There was no significant difference in the change of the Stroop word test and colour test among the three groups from baseline to week 4. For the Stroop Colour-Word Test, a statistically significant time effect (*F* = 3.411, *p* = 0.029, η^2^ = 0.041) was identified across the three groups, but no statistically significant differences were found for the group effect (*F* = 0.892, *p* = 0.414, η^2^ = 0.022) or the time-by-group effect (*F* = 0.570, *p* = 0.709, η^2^ = 0.014) (Fig. 2C).

#### Other secondary outcomes

There was a statistically significant difference in serum BDNF among the three groups (*F* = 19.572, *p* < 0.001, η^2^ = 0.331). A statistically significant time effect (*F* = 40.633, *p* < 0.001, η^2^ = 0.340) as well as a statistically significant time-by-group effect (*F* = 8.589, *p* < 0.001, η^2^ = 0.179) were also observed. The changes in serum BDNF (*F* = 22.813, *p* < 0.001, η^2^ = 0.366) from baseline to week 8 were significantly different among the three groups (Fig. 1C and Table 2). Post hoc analysis revealed that the CAE group (*p* < 0.001) and AE group (*p* < 0.001) showed significantly increased serum BDNF at week 8 than the control group, and no significant difference was found between the CAE and AE groups in terms of change in serum BDNF at week 8. The changes in serum BDNF from baseline to week 4 (*F* = 6.564, *p* = 0.002, η^2^ = 0.142) and from baseline to week 12 (*F* = 3.753, *p* = 0.028, η^2^ = 0.087) were significantly different among the three groups (Fig. 1C). Post hoc analysis revealed that the CAE group showed significantly increased serum BDNF at week 4 (*p* < 0.003) and week 12 (*p* < 0.026) than the control group. The AE group had a more significant increase in serum BDNF than the control group (*p* < 0.036) at week 4. No significant difference was found between the CAE and AE groups in terms of change in serum BDNF at weeks 4 and 12 (Fig. 1C).

**Fig. 3:**
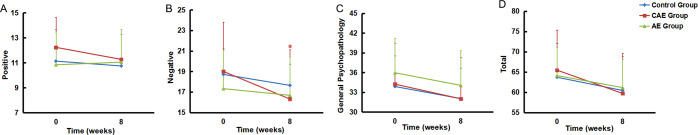
Changes in the PANSS scores (*n* = 82). **A** Changes in the positive scores; **B** changes in the negative scores; **C** changes in the general psychopathology scores; and **D** changes in the total scores. The results are expressed as mean ± SD. Differences between groups were analysed using a two-way ANOVA for repeated measures, and post hoc multiple comparisons at different endpoints were performed using the Bonferroni–Dunn test. *p ≤ 0.05, as compared with the control group. AE aerobic exercise, CAE CCRT combined with aerobic exercise.

Changes in PANSS negative scores from baseline to week 8 were significantly different among the three groups (*F* = 4.402, *p* = 0.018, η^2^ = 0.096) (Fig. 3B and Table 2). Post hoc analysis revealed that the CAE group showed significant improvement in PANSS negative scores than the control group (*p* = 0.015). Neither the CAE group nor the control group showed a significant difference when compared with the AE group. Meanwhile, no significant difference was found among the three groups in terms of changes in PANSS positive scores (*F* = 2.044, *p* = 0.887, η^2^ = 0.049), PANSS general psychopathology scores (*F* = 0.081, *p* = 0.922, η^2^ = 0.002), and PANSS total scores (*F* = 2.306, *p* = 0.106, η^2^ = 0.055) from baseline to the end of the intervention (Fig. 3A, C, D and Table 2).

#### Correlations between cognitive performance and other secondary outcomes

Changes in PANSS negative scores were negatively correlated with some changes in cognitive performance (TMT-A: *r* = 0.490, *p* < 0.001; SCT: *r* = –0.533, *p* < 0.001; Stroop word test: *r* = –0.349, *p* = 0.001; Stroop colour test: *r* = –0.443, *p* < 0.001). We did not find any significant correlations between changes in other PANSS scores and cognitive performance changes. No significant relationships were found between changes in serum BDNF levels and cognitive performance changes.

#### Safety outcomes

None of the participants reported serious adverse events. Adverse effects reported during the intervention were mild and tolerable; participant-reported adverse effects included leg soreness, tiredness, headache, asthenopia, palpitations, and insomnia, and there were no statistically significant differences in terms of side effects across the three groups (Table 3).

**Table 3:** Adverse events in the present study (*n* = 82).

Adverse events	Control subjects	CAE subjects	AE subjects	*χ*^2^ value	*p* value
	(*n* = 31)	(*n* = 26)	(*n* = 25)		
Leg soreness	0	2 (7.69%)	3 (12.00%)	3.65	0.161
Tired	1 (3.23%)	5 (19.23%)	4 (16.00%)	3.869	0.144
Headache	2 (6.45%)	1 (3.85%)	1 (4.00%)	0.267	0.875
Asthenopia	3 (9.68%)	1 (3.85%)	2 (8.00%)	0.734	0.693
Palpitations	3 (9.68%)	6 (23.08%)	5 (20.00%)	2.011	0.366
Insomnia	4 (12.90%)	4 (15.38%)	2 (8.00%)	0.672	0.714

*AE* aerobic exercise, *CAE* CCRT combined with aerobic exercise.

## Discussion

In the present study, we explored the efficacy of a short-term aerobic exercise intervention (administered alone or in combination with CCRT) on cognitive function in patients with chronic schizophrenia. In contrast to the findings of previous similar studies^31,42^, our results showed that eight weeks of aerobic exercise (administered five times a week) did not statistically significantly improve processing speed or cognitive flexibility in chronic schizophrenia patients. This is consistent with the findings of two randomised controlled trials conducted in patients with dementia or Alzheimer’s disease^43,44^. Moreover, the results of a prior meta-analysis also showed that, although aerobic exercise rehabilitation statistically significantly improved working memory, social cognition, and attention/vigilance in patients with schizophrenia, it had no statistically significant impact on processing speed, verbal memory, visual memory, reasoning, or problem-solving^45^.

Although the most effective scheme for aerobic exercise training in patients with schizophrenia has not been determined^46^, the pattern of the aerobic exercise intervention applied in this study is very similar to the practice (i.e., standard of care) of exercise rehabilitation in psychiatric departments throughout China. Therefore, our results have significant clinical value. It is also important to note that previous studies have suggested that the duration of aerobic exercise may have a very large impact on cognitive improvement^47^. In addition, we note that the results of this study may have been affected by many factors, such as a short duration of exercise and study-specific differences in exercise patterns and intensities.

This study also found that short-term aerobic exercise combined with CCRT statistically significantly improved negative symptoms, processing speed and partial cognitive flexibility (i.e., without interference) in patients with schizophrenia, and our results also showed that some cognitive improvement was correlated with the remission of negative symptoms. Cognitive impairment is reportedly closely related to negative symptoms in patients with schizophrenia, and their occurrence and progression may have the same biological and pathological mechanisms^48^. Moreover, there are many items about negative symptoms in PANSS involving cognitive flexibility and abstract thinking. Therefore, it is not difficult to comprehend the results of this study. Unfortunately, the CCRT group was evaluated on its own due to limitations in the number of participants included in the present study. However, previous studies have shown that CCRT improves cognitive function in patients with schizophrenia^49^. Therefore, it is impossible to tell whether the improvement in cognitive function seen in this study could be completely attributed to CCRT treatment. Meanwhile, we also found that differences in performance in the Stroop word-colour test were not statistically significant among the three groups, which indicates that the effect of aerobic exercise combined with CCRT on the ability to inhibit cognitive interference^50^ and on other complicated cognitive functions may be less than expected.

Our findings also suggest that, although short-term aerobic exercise may not improve cognitive performance, it may increase serum BDNF levels in patients with schizophrenia (as seen in the present study). This finding is somewhat easier to explain, as previous studies have shown that aerobic exercises such as jogging can increase expression levels of serum BDNF by increasing the synthesis and accumulation of neuroactive metabolites (e.g., actin and ketone bodies)^30,51,52^. However, in the present study, we did not find a significant correlation between the change in serum BDNF level and the improvement of cognitive function. BDNF in serum consists of BDNF bound to platelets and BDNF circulating freely (unbound) in plasma. Since the BDNF stored in platelets is more than 100 times that in plasma, the vast majority of BDNF in serum may not be able to freely pass through the blood–brain barrier to affect the brain function^53^. This may also be one of the explanations for why aerobic exercise was not found to improve cognitive brain function in this study.

Our study also found that the combination of CCRT and aerobic exercise did not statistically significantly increase serum BDNF levels induced by aerobic exercise. There are two possible explanations for this phenomenon. First, previous studies have shown that the effect of cognitive training on serum BDNF levels is relatively limited^54^, and that short-term CCRT is not sufficient to meaningfully increase serum BDNF levels. Second, this finding may be due to the possibility that aerobic exercise might increase serum BDNF to a certain level and that CCRT will not further meaningfully increase BDNF expression. Hence, we plan to enrich this evaluation with a comparative evaluation conducted with the enrolment of a healthy control group as well as a group treated with CCRT alone in future research efforts.

This study has some limitations that need to be noted. First, although the sample size of this study was larger than the minimum theoretical sample size, it was still limited. And in consideration of the ability to participate, most of the patients included in this study only suffered from mild to moderate cognitive impairment, which may also limit the promotion of the results. Secondly, we did not set up an intervention evaluating CCRT alone, and hence we could not clarify the independent effect of CCRT. Third, the single-blind design may have led to some bias in the results. In addition, BDNF circulating freely in plasma, which can cross the blood–brain barrier, may be more suitable as a biological marker of brain function than serum BDNF. Finally, the short duration of the intervention may limit the recorded effect of aerobic exercise.

## Conclusions

Our study suggests some cognitive performance metrics (i.e., processing speed rather than the ability to inhibit cognitive interference) in patients with schizophrenia may be improved in a short period of time when aerobic exercise is combined with CCRT. Short-term aerobic exercise may increase serum BDNF levels in patients with schizophrenia. However, increased serum BDNF does not necessarily indicate an amelioration of cognitive performance. Our findings, which should be confirmed in future highly powered studies enrolling healthy controls and including patients administered CCRT alone, provide preliminary evidence for improving interventions in schizophrenia patients.

## Data Availability

The data that support the findings of this study are available from the corresponding author upon reasonable request.
